# A Compound Composite Odontoma Associated with Unerupted Permanent Incisor: A Case Report

**DOI:** 10.5005/jp-journals-10005-1030

**Published:** 2009-08-26

**Authors:** Usha Mohan Das, Deepak Viswanath, Umme Azher

**Affiliations:** 1 Principal, Professor and Head, Department of Pedodontics and Preventive Dentistry, VS Dental College and Hospital KR Road, VV Puram, Bengaluru-560004, Karnataka, India; 2 Assistant Professor, Department of Pedodontics and Preventive Dentistry, VS Dental College and Hospital, KR Road VV Puram, Bengaluru-560004, Karnataka, India; 3 Senior Lecturer, Department of Pedodontics and Preventive Dentistry, VS Dental College and Hospital, KR Road, VV Puram Bengaluru-560004, Karnataka, India

**Keywords:** Compound odontoma, odontogenic tumor, unerupted incisor.

## Abstract

Odontomas are the most common type of odontogenic tumors and are generally asymptomatic. Frequently they interfere with the eruption of the teeth. This is the case report of a compound composite odontoma in an 11 years old girl, which resulted in failure of eruption of the permanent maxillary right central incisor while the contralateral tooth had erupted. A calcified mass was seen in the radiograph and was provisionally diagnosed as odontoma following which the odontoma was enucleated. Routine follow-up was done for 1½ years and no recurrence was seen.

## INTRODUCTION

Odontomas are considered to be developmental anomalies resulting from the growth of completely differentiated epithelial and mesenchymal cells that give rise to ameloblast and odontoblast. These tumors are basically formed of enamel and dentin but they can also have variable amounts of cementum and pulp tissue.^1^ Like teeth, once fully calcified they do not develop further.^2^ Even when the morphology is grossly distorted (as in complex odontomas) the pulp,dentine, enamel and cementum are in normal anatomical relationships with one another and also like teeth, odontomas may erupt.

Paul Broca was the first person to use the term ‘Odontoma’ in 1867. He defined the term odontoma as "tumors formed by the overgrowth of transitory or complete dental tissues". Odontomas are hamartoma arising during normal tooth development, and they often reach a fixed size and are composed of mature enamel, dentine, cementum and pulp tissues.^3^

During the development of the tumor, enamel and dentin can be deposited in such a way that the resulting structures show an anatomic similarity to normal teeth in which the lesion is classified as compound odontoma. However, when the dental tissues form a simple irregular mass occurring in a disorderly pattern, it is described as a complex odontoma.^4^ Compound odontomas occur more frequently than complex odontomas.^5^,^6^

## CLASSIFICATION (BY WHO)

1. *Complex odontoma:* A malformation in which all the dental tissues are well-formed but occurring in a less orderly pattern.
2. *Compound odontoma:* A malformation in which all the dental tissues are arranged in a more orderly pattern than in the complex odontoma, so that the lesion consists of many tooth like structures.

These odontogenic tumors may be found anywhere in the dental arches. The majority of odontomas which are located in the anterior region of the maxilla are compound, while the great majority of odontomas located in the posterior areas, especially in the mandible, are complex odontomas.^1^,^7^,^8^

The etiology of the odontoma is unknown.^3^ However, it has been suggested that trauma and infection at the place of the lesion can offer ideal conditions for its appearance. In general, they are asymptomatic, have slow growth and seldom exceed the size of a tooth, but when large can cause expansion of the cortical bone.^2^

Hitchin suggested that odontomas are either inherited or are due to a mutant gene or interference, possibly postnatal, with the genetic control of tooth development. On the other hand, Levy has reported the experimental induction of this lesion in rat by traumatic injury.^9^

Although the etiology of this malformation is not yet known, there is some evidence to show that there is genetic basis for both complex and compound composite odontomas. Heredity is a possible factor and persistent lamina could be the hidden inherited developmental anomaly. The mystery of the cytogenic changes in tooth development and how these changes are controlled by DNA molecules in nuclei remains unsolved and much research is still to be done.^10^

Odontomas of all types comprise approximately 22% of odontogenic tumors of jaws.^11^ There is no gender predilection and an odontoma can occur at any age but most commonly occurs in the second decade of life.^12^ One study analyzed 396 cases and showed that diagnosis usually happens between ages 11 and 15 years.^6^ Another study comprising 149 cases concluded that the lesions are detected most often during the second decade of life.^8^ Many times odontomas are found associated with unerupted teeth.^1^,^8^,^13^-^18^ The canines, followed by upper central incisors and the third molars, are the most frequent teeth impacted by odontomas. In very few instances odontomas are related to missing teeth. Generally these malformations are intraosseous, but may erupt into the oral cavity.^18^,^19^

Radiographic aspects of odontoma are characteristic. The complex odontoma appears as an irregular mass of calcified material surrounded by a thin radiolucent area with smooth periphery and the compound odontoma type shows calcified structures resembling teeth in the center of a well defined radiolucent lesion. A periodontal or pericoronary space characteristic of unerupted teeth is seen around each tooth.^3^,^5^ A developing odontoma may be discovered by routine radiography, but may cause difficulty in identification due to lack of calcification.^3^

The histological examination often shows the presence of enamel matrix, dentin, pulp tissue, and cementum that can, but need not exhibit normal relationship.^1^,^3^Compound odontomas are formed by tooth-like structures which resemble pulp tissue in the central portion surrounded by a dentin shell and partially covered by enamel. Complex odontomas are conglomerates without orientation of dentin, enamel; enamel matrix, cementum and areas of pulp tissue. Connective tissue capsule surrounding an odontoma is similar to the follicle that covers a normal tooth.^3^

Odontomas are treated by conservative surgical removal and there is little probability of recurrence.^1^,^19^ Ameloblastic fibro-odontomas and odontoameloblastomas show a great resemblance to common odontomas, especially in the radiographic examination. Therefore it has been suggested that all specimens be sent to an oral pathologist for microscopic examination.^3^,^19^ Besides, proper patient care should include careful clinical and radiographic follow-up.^20^

## CASE REPORT

**Fig. 1:**
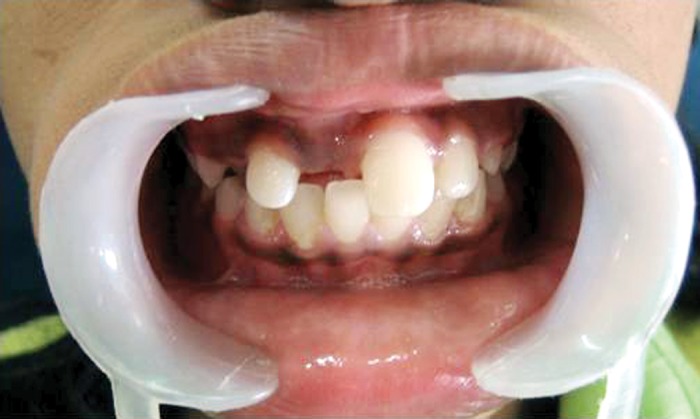
Unerupted permanent maxillary right central incisor in a 11-year old patient

**Fig. 2:**
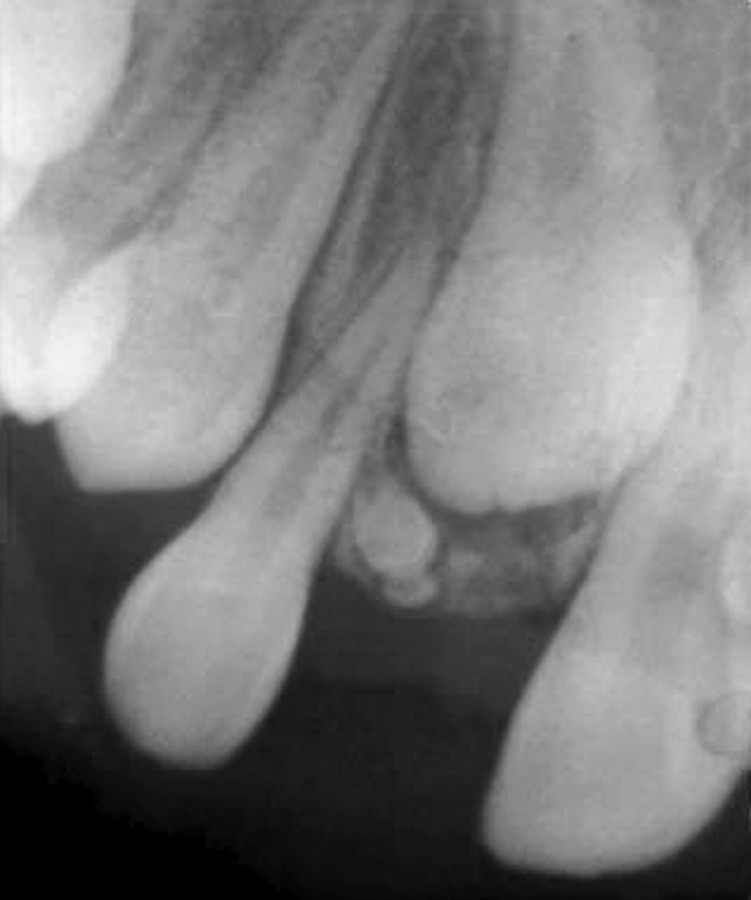
IOPA radiograph showing the presence of a radiopaque mass in the region of unerupted tooth

**Fig. 3:**
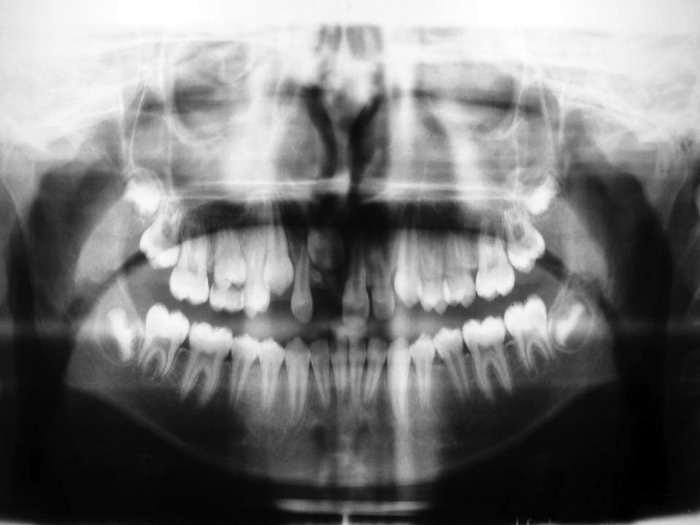
Orthopantomograph showing the presence of a radiopaque mass in the region of unerupted tooth

A 11 years old female patient reported to the Department of Pedodontics and Preventive Dentistry, VS Dental College and Hospital, Bengaluru with a chief complaint of unerupted upper right front tooth, while the contralateral tooth had already erupted. Clinical examination revealed no facial asymmetry extraorally. Intraorally, unerupted 11 without any swelling or inflammation of the overlying mucosa was noticed (Fig. 1). The intraoral periapical (Fig. 2), and panoramic radiographs (Fig. 3) revealed the presence of a radiopaque mass in 11 region obstructing its eruption. Medical and family history was noncontributory.

**Fig. 4:**
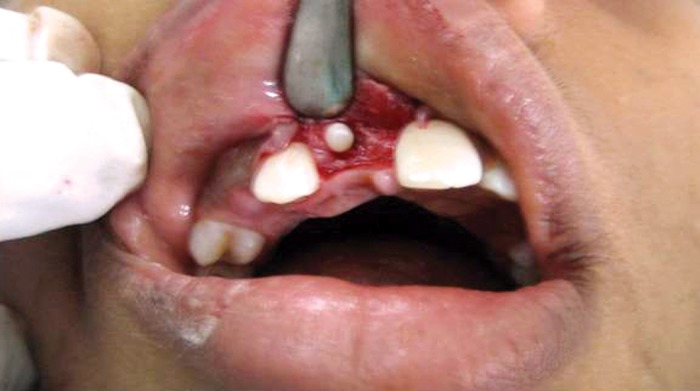
Enucleation of the odontome

**Fig. 5:**
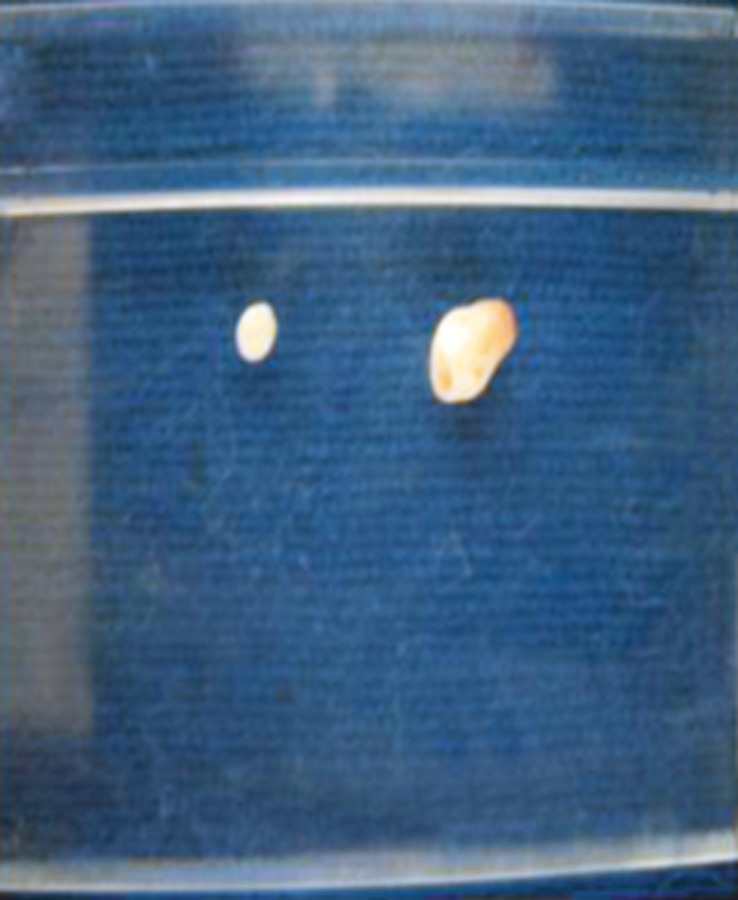
The calcified mass which was removed surgically

**Fig. 6:**
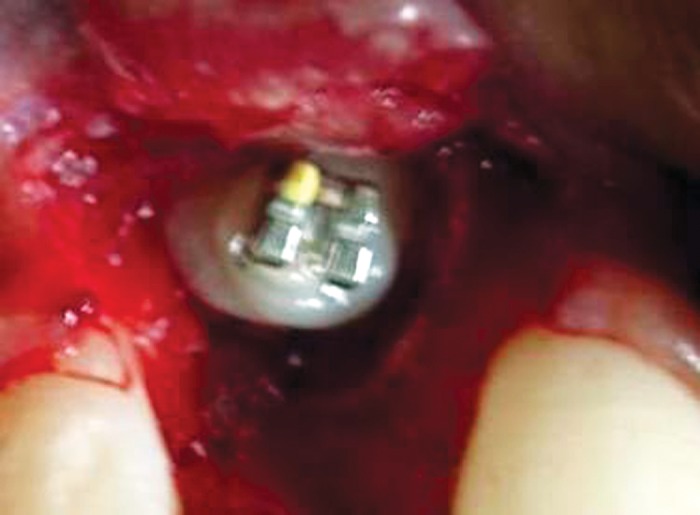
An orthodontic bracket was bonded to the labial surface of the incisor crown

**Fig. 7:**
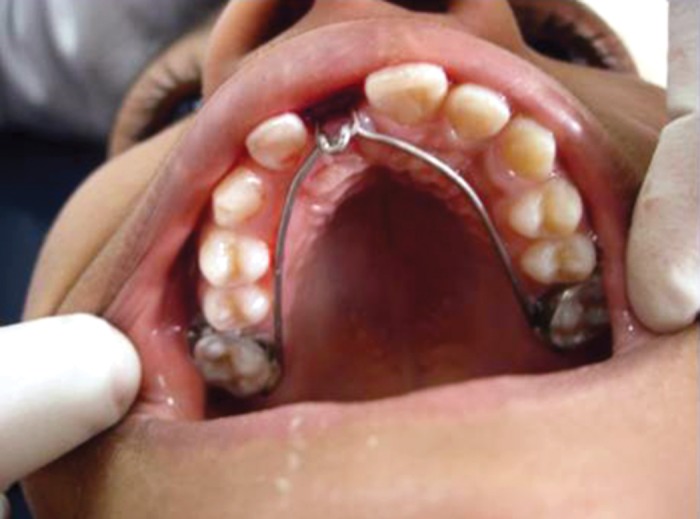
A palatal arch with a hook was cemented to maxillary first permanent molars

**Fig. 8:**
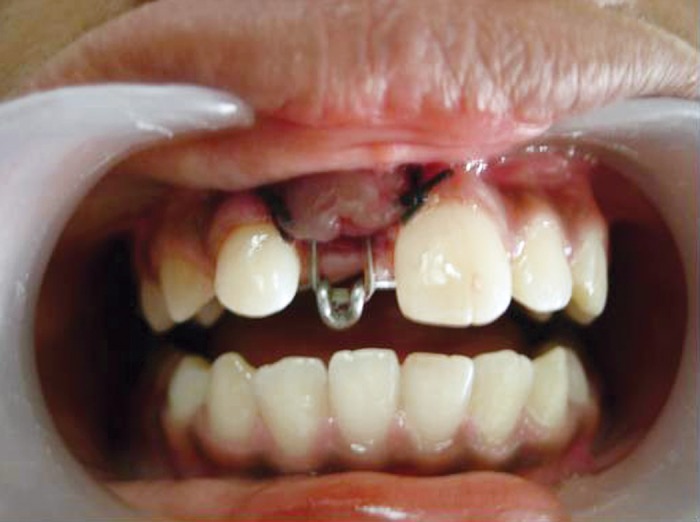
Traction force was applied on the tooth using orthodontic elastics

On the basis of clinical and radiographic findings, it was provisionally diagnosed as an odontoma. Clark’s radiographic technique revealed the presence of two radiopaque masses in the labial region of the upper right central incisor. Under local anesthesia, surgical removal was done. A mucoperiosteal flap on the labial surface from the right permanent central incisor to left permanent lateral incisor was reflected. The layer of bone overlying the labial surface was removed and the calcified mass was exposed (Fig. 4). The calcified mass was removed without disturbing the underlying tooth and sent for histopathological examination (Fig. 5). After homeostasis, the area was irrigated and the tooth was exposed. As the impacted permanent tooth showed a fully formed root, little spontaneous eruption could be expected. Therefore it was decided to place an orthodontic device in order to guide the impacted tooth into its position. At the time of surgery, a bracket was bonded to the labial surface of the incisor crown (Fig. 6). The surgical flap was apically positioned and sutured in place. A palatal arch with a hook was later fixed to maxillary first permanent molars in order to achieve a better control of the movement of the tooth (Fig. 7). Traction force was applied on the tooth using orthodontic elastics (Fig. 8).

**Fig. 9:**
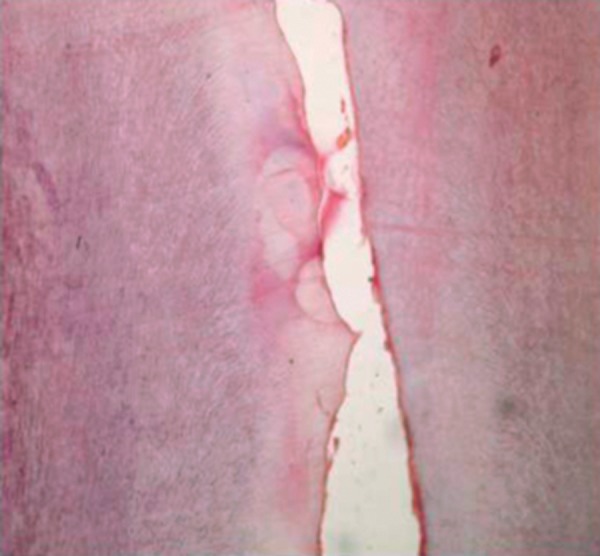
Photomicrograph of the decalcified section of the odontoma showing dentinal tubules

The histopathological examination confirmed the provisional diagnosis of compound composite odontoma (Fig. 9). The decalcified specimen showed dentin in cross and longitudinal sections. The dentin appeared to have normal tubular pattern. Strands of connective tissue, empty space of pulp chamber were also seen.

**Fig. 10:**
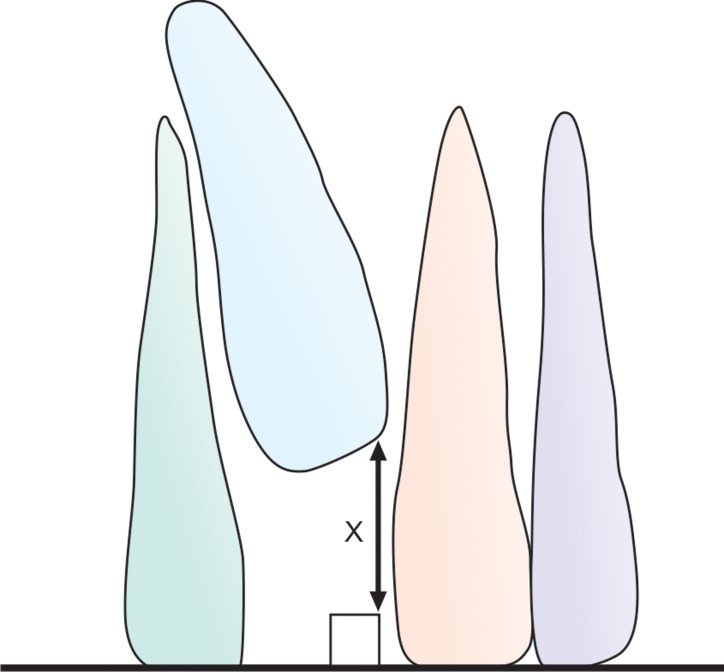
The position of the impacted tooth was determined on IOPA radiograph by drawing a perpendicular line from the most apical point on the incisal edge of the impacted tooth

Patient was advised a review appointment once in 3 months in order to assess the eruption of unerupted tooth and to examine the recurrence of the odontoma. The position of the impacted tooth was determined on IOPA radiograph by drawing a perpendicular line from the most apical point on the incisal edge of the impacted tooth to a line connecting the incisal edges of the adjacent nonimpacted teeth (Fig. 10). The length was measured in millimeters.

Patient was followed up for 1½ years and the permanent maxillary right central incisor was observed to move down along the path of eruption without any evidence of recurrence of the bony mass.

**Fig. 11:**
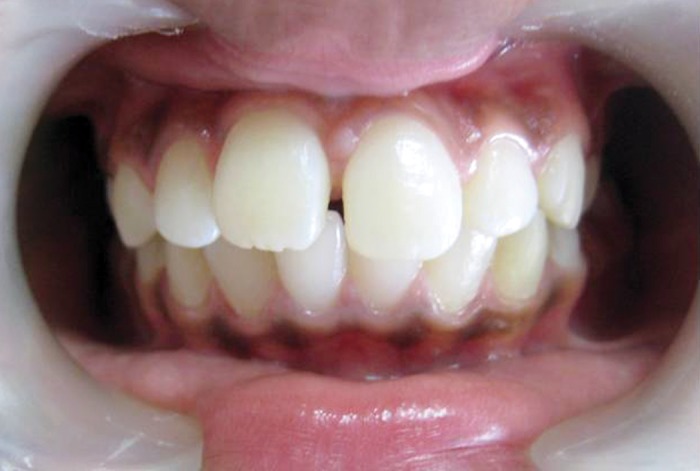
The permanent maxillary right central incisor in its position after the treatment

The protocol followed was clinical and radiographic examinations every six months and clinical examination alone in other visits. After 1½ years, the permanent maxillary right central incisor was finally brought into the dental arch (Fig. 11). Clinical and radiographic examination revealed no soft tissue or osseous defect.

## DISCUSSION

Odontomas are relatively common odontogenic lesions, generally asymptomatic, and are rarely diagnosed before the second decade of life. They frequently lead to impaction or delayed eruption of permanent teeth.

Radiographic aspects of odontoma are characteristic. The complex odontoma appears as an irregular mass of calcified material surrounded by a thin radiolucent area with smooth periphery. Compound odontoma type shows calcified structures resembling teeth in the center of a well defined radiolucent lesion. Periodontal or pericoronary space characteristic of unerupted teeth is seen around each lesion.

A developing odontoma may be discovered by routine radiography, but may cause difficulty in identification due to lack of calcification.

In the absence of proven facts related to etiology, classification must be made on a morphological process concerned in the production of supernumerary tooth and a compound composite odontoma may be identical. But it is evident that there are three main types of compound composite odontomas:

1. *Denticular type:* Composed of two or more separate denticles, each having a crown and a root or epithelial sheath of hertwig with a distribution of dental hard tissue comparable to that found in a tooth.
2. *Particulate type:* Composed of two or more separate masses or particles bearing no macroscopic resemblance to a tooth and consisting of hard dental tissues abnormally arranged.
3. *Denticulo particulate type:* Denticles and conglomerate masses or particles are present side by side.

Literature suggests that odontoma once enucleated usually does not recur but in young children, a close monitoring is necessary. Early removal of the cause of eruption disturbances is important in the developing dental arch. In addition, a careful follow-up review of the case both clinically and radiographically to assess the eruption of the unerupted or impacted teeth is necessary.

## CONCLUSION

Clinical experience suggests and the dental literature supports that an individualized radiographic examination of any pediatric patient who presents clinical evidence of delayed permanent tooth eruption or temporary tooth displacement with or without a history of previous dental trauma should be performed. Early diagnosis of odontomas allows adoption of a less complex and less expensive treatment and ensures better prognosis.
